# Supplementary figures and images for: DNA Specificity Determinants Associate with Distinct Transcription Factor Functions
Source: PLoS Genet. 2009 Dec 18;5(12):e1000778. doi: 10.1371/journal.pgen.1000778 (PMC2787013; doi:10.1371/journal.pgen.1000778)

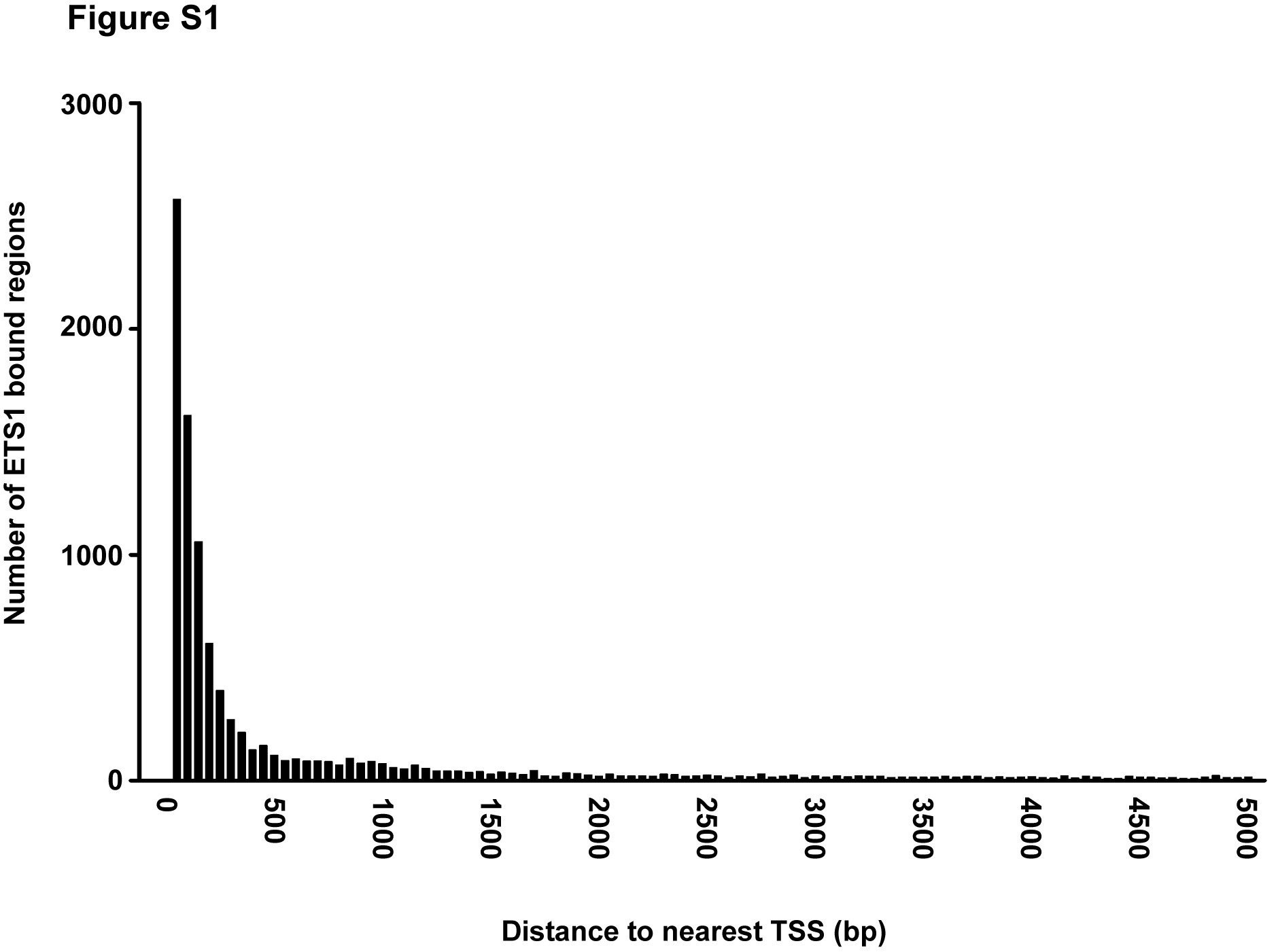

Supplement: Figure S1 — ETS1 bound regions have a high density within 500 bp of a TSS. The distance from the center of each of the 19,420 ETS1 bound regions to the nearest Refseq TSS was recorded. Distances were binned in 50 bp bins and frequencies plotted for bins from 0 to 5,000. Distances greater than 5,000 were discarded. 7,137 ETS1 bound regions had centers within 500 bp of a TSS. (2.64 MB TIF) [file pgen.1000778.s001.tif]

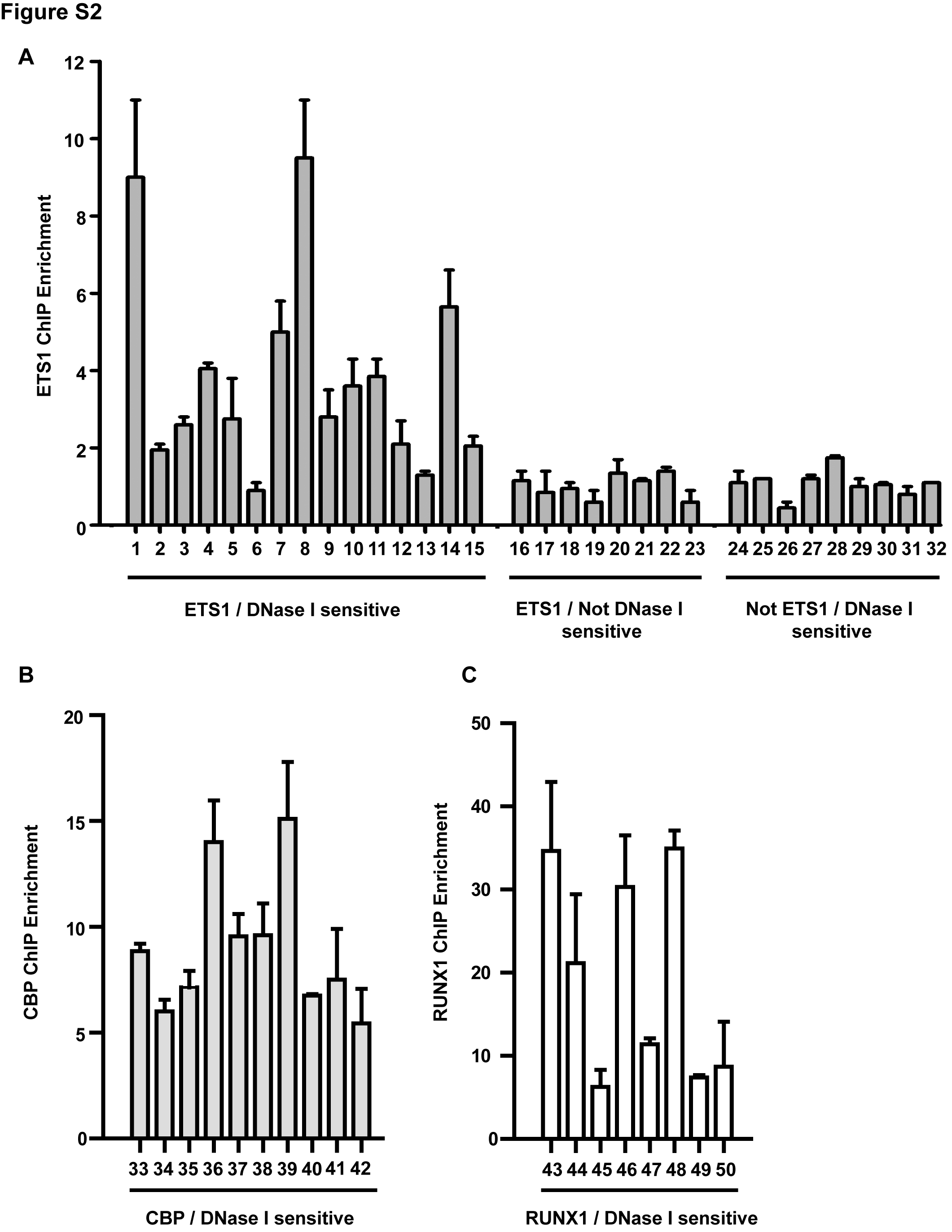

Supplement: Figure S2 — Validation of ChIP-seq results by Q-PCR analysis of ChIP DNA. Enrichment was tested using primer sets specific for each region (Table S1). The level of each region was determined by comparison to a standard curve of ChIP input DNA. Enrichments are a ratio of the level of the target region in each sample over the mean of the level of two negative control genomic regions. The enrichments shown are the mean and standard error of the mean of two independent ChIP experiments. Regions were considered bound that had an mean enrichment of equal to or greater than 2. (A) 15 randomly selected ETS1 bound regions that overlapped with a DNase I sensitive site in CD4+ T cells (1–15), 8 randomly selected ETS1 bound regions that did not overlap with a DNase I sensitive site (16–23), and 9 randomly selected DNase I sensitive regions that were not scored as ETS1 bound (24–32) were tested for ETS1 ChIP enrichment in Jurkat T cells. Significantly more (13 of 15) ETS1 bound/DNase I sensitive regions were verified compared the other two categories to (0 of 8 and 0 of 9; P<0.0001, Fisher's exact test). (B) 10 of 10 randomly selected CBP bound/DNase I sensitive regions were verified for CBP enrichment. (C) 8 of 8 randomly selected RUNX bound/DNase I sensitive regions were verified for RUNX enrichment. (5.85 MB TIF) [file pgen.1000778.s002.tif]
