# Supplementary material for: DNA Specificity Determinants Associate with Distinct Transcription Factor Functions
Source: PLoS Genet. 2009 Dec 18;5(12):e1000778. doi: 10.1371/journal.pgen.1000778 (PMC2787013; doi:10.1371/journal.pgen.1000778)
Supplement: Table S2 — Position weight matrixes for Motif 1, 2, and 4. (0.12 MB DOC) [file pgen.1000778.s004.doc]

| Motif 1 | A | 105 | 23 | 43 | 0 | 0 | 278 | 279 | 28 | 23 | 53 | 84 |  |  |  |  |  |  |
| --- | --- | --- | --- | --- | --- | --- | --- | --- | --- | --- | --- | --- | --- | --- | --- | --- | --- | --- |
|  | C | 33 | 185 | 234 | 0 | 0 | 2 | 0 | 9 | 55 | 33 | 82 |  |  |  |  |  |  |
|  | G | 128 | 72 | 3 | 280 | 280 | 0 | 1 | 243 | 20 | 163 | 100 |  |  |  |  |  |  |
|  | T | 14 | 0 | 0 | 0 | 0 | 0 | 0 | 0 | 182 | 31 | 14 |  |  |  |  |  |  |
| Motif 2 | A | 13 | 0 | 1 | 0 | 2 | 87 | 41 | 23 | 0 | 0 | 0 | 92 | 0 | 0 | 2 | 5 | 11 |
|  | C | 60 | 14 | 0 | 0 | 0 | 1 | 11 | 15 | 6 | 0 | 2 | 0 | 0 | 0 | 39 | 46 | 30 |
|  | G | 13 | 0 | 95 | 96 | 93 | 6 | 36 | 8 | 3 | 96 | 4 | 4 | 96 | 0 | 1 | 3 | 12 |
|  | T | 10 | 80 | 0 | 0 | 1 | 2 | 8 | 50 | 87 | 0 | 90 | 0 | 0 | 96 | 54 | 42 | 43 |
| Motif 4 | A | 14 | 42 | 97 | 0 | 23 | 0 | 0 | 0 | 0 | 6 | 12 |  |  |  |  |  |  |
|  | C | 116 | 87 | 25 | 128 | 0 | 0 | 168 | 177 | 19 | 55 | 75 |  |  |  |  |  |  |
|  | G | 13 | 0 | 37 | 0 | 0 | 13 | 0 | 0 | 0 | 75 | 14 |  |  |  |  |  |  |
|  | T | 34 | 48 | 18 | 49 | 154 | 164 | 9 | 0 | 158 | 41 | 76 |  |  |  |  |  |  |
