# Supplementary material for: DNA Specificity Determinants Associate with Distinct Transcription Factor Functions
Source: PLoS Genet. 2009 Dec 18;5(12):e1000778. doi: 10.1371/journal.pgen.1000778 (PMC2787013; doi:10.1371/journal.pgen.1000778)
Supplement: Table S3 — Oligonucleotide primers used in this study. (0.07 MB DOC) [file pgen.1000778.s005.doc]

| Region number | Region coordinates | 5’ primer | 3’ primer |
| --- | --- | --- | --- |
| 1 | chrX:128867633-128867882 | GAAGTGACGTGTACGTCGCG | ACATGGACCAAGAACGGAAGC |
| 2 | chr9:119628109-119628353 | GAGGTATGTTAGGTGTAACAGC | CTGGCAGCCACAGTCACC |
| 3 | chr6:31273353-31273599 | GCAGCAGCAGGAAGAGTTTCC | ACGACGGATTCGGAGACAGG |
| 4 | chr4:170770236-170770454 | GGACAACTGCTCGAGAGGC | CCGCTCTCCCTTAGTCCG |
| 5 | chr3:16328541-16328788 | CTTAGTCTAGCCACTGAAACG | GCTAGGTTTCTGAGGCAGC |
| 6 | chr20:39201112-39201362 | GAGGAAGGAAGGTCTTGTGG | GGCTGCCATTTCTAAGTTGAG |
| 7 | chr2:61969169-61969420 | CGGTAAGCCCTCACTGCC | GACAGCCTCCTACGTCACG |
| 8 | chr3:142939530-142939781 | CGCAGCAGGCAGATTCAGC | CACAGTGACGTCACCTGACG |
| 9 | chr6:139487847-139488089 | CAGCTTTGTACACCAGAGTGC | GTTGCATGCAGATACAGATACC |
| 10 | chr8:145674103-145674309 | GGAAGTGTAGCGTTGCCATGG | GTGGGTCCGCTGGCTTCC |
| 11 | chr1:227140863-227141110 | GCTCTGTGAACAGGAAGAGG | CAGCCCCTCCACTTCAGAGC |
| 12 | chr8:53037425-53037672 | CCAGGCAAGCGTCACTCACC | GACAGAGGTGCAATTGGCAACAG |
| 13 | chr17:78415237-78415470 | GGCTGCCATCTGTGTTGTGG | CCCTGGAAGCACAAGTGAAGC |
| 14 | chr20:56659554-56659802 | GCGAGCAAGGCTTGCGAGC | CTGTGGTCCCGGGTTCTGG |
| 15 | chr5:53641915-53642162 | AGATCCGAGAGGCGACATGC | GCCGGATGTCTGATCTCCG |
| 16 | chr12:121206236-121206483 | CTGCTGCTGATATCAAGTGCG | GCTGTGTGCTATGATAGATGAC |
| 17 | chr10:98859442-98859669 | CTCTCAGTCCCTGCCAAGC | GGAGGTTTGCAAGAGAAGAGC |
| 18 | chr1:156509386-156509624 | CACCCTTTGAGCTGATTGTGG | GAAGTGAGCACATCGACTTCC |
| 19 | chr5:145157136-145157384 | GCTAACTGTGCATCCAACATCC | GTAACAAGCCACCATGCTTGC |
| 20 | chr15:76241474-76241724 | GTGATTAGTCATGCTAGCTGGC | GGAAAGAGTGTGACAGCAACG |
| 21 | chr19:34714888-34715135 | AAGGGCTAGGTCTAGAAGTGG | CCAAGGACTACATTTCCCAGC |
| 22 | chr18:11567750-11567971 | GGCCTGTTTCTGATTCTACG | CTGCCTACTTTCACAGACCC |
| 23 | chr18:682883-683131 | CAACACTCTTCAAGGCCATGG | AGTGCAACACCTGGGTGGC |
| 24 | chr3:90332580-90332980 | GTAGTACCTGGTGGCAATTGG | GCTCAGAAGGTGAAGAGAAGC |
| 25 | chr18:44460021-44460091 | GACCACATCAGCCCACAGAGG | GGAGGCACAAATGCATGGAGG |
| 26 | chr1:145987245-145987385 | GAGAAGATAAAACCTTCCCTGC | CGATCACTAGAGAATCTTCCC |
| 27 | chr2:230408591-230409531 | GGAAGTATATGCCTGTATCTAAACC | GACATTTCTCAATGACATGAACAACTG |
| 28 | chr2:53994439-53994669 | CCGTGGAGTAGTAACTGTTTCC | GGGCTAGCTAACCTCTCTTGG |
| 29 | chr1:112091485-112091626 | GGTTATGCATAGTGACCAAACCC | CAAACAACTGGAAATACCACCAGG |
| 30 | chr9:113493887-113494287 | GAACGGGTGATTCTCTTGTTCC | CGGACCCTATCTTTTCACTAGAGG |
| 31 | chr2:230827074-230828374 | GGAACTGAACACCTCACAGTGG | GAGCAAACAGGACATTCATCCC |
| 32 | chr9:128954055-128954812 | GCTTCCCTCTGAGCACACTCC | GGTTCTAGAATGCTCTGACTCAC |
| 33 | chr4:45767842-45767932 | GGCCATTCTAACGTCCTAGC | TTTTAAACAACTGGCAGCATAATAGG |
| 34 | chr12:127761573-127761622 | GCGCGTATTTGTGTGTAGGG | TGGGCGCAGCCAATGCC |
| 35 | chr12:6423123-6423170 | GGAATTCAGGTGCCTTGCC | CTCTGCAGCTCTTCATGGG |
| 36 | chr8:104417987-104418035 | CAGAAACTTACCACAGGTGGG | TTCATTTCCTGAGAGTAACTGCC |
| 37 | chr2:98740929- 98740974 | CTGACAGTGCCAGGAACGC | GCAGCCAATAGCCCAGCC |
| 38 | chr6:236803-236835 | TCGGTCTTGGTGCATGGG | ATCAGGTGCTAATCAGAGGCC |
| 39 | chr11:106833232-106833789 | TGAAGTGAGCAGTATCATTGCC | CTTATGGCTTATGACTAGGTTCC |
| 40 | chr3:114207352-114207403 | AAACTCCACTATTCAAGAGCTGC | TACAGATACCAGTGCTAAGTGG |
| 41 | chr12:4797385-4797591 | ACGCAGTCTGCTGGCGC | CCCTCCCAGCTGCAGG |
| 42 | chr6:111516256-111516303 | TGTGTTTGCAAGCAGGTCGC | ACTGGGGTTGAAGGTTCTGG |
| 43 | chr13: 112575387- 112575629 | AGCGGTGTGAGGTGTGGC | AAAGCCACACCTCACACCG |
| 44 | chr14:105518923- 105519173 | ACAACAGTCAGATTGCATTAGGG | CTTGGATTAGTGTCCTTTAAAGAGG |
| 45 | chr6:41103875- 41104125 | CTGAGGCCATCTCCTGGG | CCAGCCTCCCAGCAGC |
| 46 | chr6: 45231940- 45232188 | CACAGAATACAGCCACCTGG | CTATACAAATGTTGAGGATATCTGC |
| 47 | chr7: 30336954- 30337198 | TCTAAGTTATCGCTGCTTGAGC | AAAACTCAACCACAGAAACCACC |
| 48 | chr4:776747- 776982 | GTGTGCACCACGTGGTCG | CAACCACAAGACGACAAGCC |
| 49 | chr7: 35731452- 35731683 | CAGTTGTCCGGAGCTCCC | CGACAGATGTGCGGCTCG |
| 50 | chr8: 22931989- 22932240 | TCCGAGGGCAACACAGGG | CTGCAGTCTCCAGATGGGC |
| RPS26 | chr12:054722044-054722104 | CAGCAGAAATGCTGAATGTAAAGG | CATGAGATCCCTACGCGGAC |
| TCRβ | chr7:142216971-142217064 | GGAATCTAGGTATCCCAGATCC | CTGTGGCCTTTGATCTTGTGC |
